# Patients with stage II of the knee osteoarthritis most likely benefit from the intra-articular injections of autologous adipose tissue—from 2 years of follow-up studies

**DOI:** 10.1007/s00402-021-03979-w

**Published:** 2021-06-11

**Authors:** Paweł Bąkowski, Jakub Kaszyński, Cezary Baka, Tomasz Kaczmarek, Kinga Ciemniewska-Gorzela, Kamilla Bąkowska-Żywicka, Tomasz Piontek

**Affiliations:** 1 Department of Orthopedic Surgery, Rehasport Clinic, Poznan, Poland; 2 Institute of Bioorganic Chemistry Polish Academy of Sciences, Poznan, Poland; 3 Department of Spine Disorders and Pediatric Orthopedics, University of Medical Sciences Poznan, Poznan, Poland

**Keywords:** Autologous subcutaneous adipose tissue, Platelet-rich plasma, Knee OA, Intra-articular injection

## Abstract

**Background:**

Knee osteoarthritis (OA) is a common, chronic, progressive and degenerative disease which affects patients’ quality of life and may cause disability and social isolation. OA is a huge economic burden for the patient and a large strain for the whole healthcare system. Articular cartilage has a small potential to repair, with progressively more clinicians emphasizing cellular therapy. Subcutaneous fat tissue in human body is a large reservoir of mesenchymal stem cells (MSCs) and is been harvested in minimally invasive, simple procedure. The purpose of this study was to define a specific group of patients with knee osteoarthritis, who are the most likely to benefit from the treatment with intra-articular injection of an autologous adipose tissue (AAT).

**Methods:**

From 2016 to 2018, 59 symptomatic bilateral and unilateral knee OA patients were treated with a single intra-articular (IA) injection of an autologous adipose tissue (AAT). Before the treatment and at the follow-up, the participant was asked to fulfill the Knee Injury and Osteoarthritis Outcome Score (KOOS), the International Knee Documentation Committee 2000 (IKDC 2000), The Western Ontario and McMaster Universities Osteoarthritis Index (WOMAC), the Health Questionnaire EQ-5D-5L and to quantify the pain in the affected joint with a Numeric Rating Scale (NRS). Moreover, the patients were asked to: (i) assess their satisfaction with the effects of the conducted treatment: from 0 (unsatisfied) to 10 (very satisfied), (ii) describe the rehabilitation, if it was performed (supervised or individual and duration in weeks) and (iii) indicate any additional treatment applied, like IA injections of hyaluronic acid (HA) or platelet-rich plasma (PRP), knee arthroscopy, partial or total knee arthroplasty (TKA) at the follow-up.

**Results:**

The mean age of 37 participants (16 males and 21 females) included into statistical analysis was 57.78 ± 7.39 years, the mean BMI was 31.30 ± 7.51. The questionnaires were fulfilled after the average follow-up time of 27 ± 6.5 months. A significant difference (*p* < 0.05) compared with the baseline, was observed in pain [NRS], WOMAC, KOOS index, pain, symptoms, ADL, Sport and Rec, QoL, EQ-5D-5L index. The satisfaction in the whole group was 6.16 ± 3.07. There was no significant difference between satisfied and unsatisfied patients in BMI and pain [NRS] at the baseline. 6 out of 7 patients with stage IV in K-L were unsatisfied with the effects of the treatment with AAT.

**Discussion:**

The main conclusion of this study is that the patients with stage II of the knee OA with normal BMI are were most likely to benefit from IA injection of AAT, in contrast to the patients with stage IV, who will not beware not satisfied with the effectiveness of this kind of treatment. There were no adverse events reported at the donor site as well as in the treated knee joints.

## Introduction

Knee osteoarthritis (knee OA) is a slowly progressive disease which causes irreversible changes in the affected joint. It appears with the degeneration of articular (hyaline) cartilage, synovium, ligaments and menisci, subchondral bone sclerosis, osteophyte formation at joint margins, changes in joint axial alignment. Patients’ symptoms include a persistent pain, joint effusion and a limited range of motion [1, 2]. The risk factors of knee OA may be divided into three groups: genetic factors, constitutional factors (aging, obesity, female sex) and local risk factors (injury, local muscle weakness, joint laxity) [3–6]. A standardized treatment protocol for patients has not been established so far. The method is chosen according to the given patient symptoms intensity and a joint condition. Weight loss, physical therapy, nonsteroidal anti-inflammatory drugs (NSAIDs) are the first line treatment options. However, in some cases, a conservative treatment fails and more invasive procedures are then considered, including the arthroscopic (AS) debridement, the ligaments and/or articular cartilage reconstruction, the menisci repair or the total knee arthroplasty [1, 2, 7–12].

Intra-articular injections with Mesenchymal Stem Cells or, according to Arnold Caplan, Medicinal Signaling Cells (MSCs) [13–15], seem to be a promising method to preserve joints for which a conservative treatment did not stop a progression of the disease [16, 17]. It has been shown that adipose tissue is a better source of MSCs than bone marrow, due to the higher concentration of pericytes (2% *vs* 0,02%, respectively) [2]. Activated pericytes, after differentiation into MSCs, serve as a kind of “drugstore”, which decreases over-aggressive immune response and enhances regenerative processes [17–19]. However, the results of the studies aimed at the description of the AAT-MSCs influence on knee osteoarthritis are sometimes contradictory. According to the current systematic review of 18 studies, MSCs infiltrations for knee OA can represent a feasible option, leading to an overall remarkable improvement of all clinical and functional considered outcomes [20]. However, also a recent meta-analysis of five studies (220 patients) demonstrated that intra-articular MSCs have a limited evidence both in pain relief and functional improvement in knee osteoarthritis [21].

Taking into consideration all of the above, the purpose of this study was to define a specific group of patients with knee osteoarthritis, who are the most likely to benefit from the treatment with intra-articular injection of an autologous adipose tissue (AAT).

## Materials and methods

From 2016 to 2018, 59 symptomatic bilateral and unilateral knee OA patients were treated with a single intra-articular (IA) injection of an autologous adipose tissue (AAT) at Rehasport Clinic, Poznan, Poland by two experienced orthopedic surgeons (TP or PB). Firstly, every patient was qualified to this treatment procedure after a diagnostic process, which included a detailed clinical history, a physical examination and an X-ray imaging, to assess a stage of the knee degeneration with Kellgren–Lawrence scale. Patients might present bilateral knee OA in X-ray, but only one joint needed to be symptomatic to include the subject to this study.

At the day of the surgery, right before the treatment, the participant was asked to fulfill the Knee Injury and Osteoarthritis Outcome Score (KOOS), the International Knee Documentation Committee 2000 (IKDC 2000), The Western Ontario and McMaster Universities Osteoarthritis Index (WOMAC), the Health Questionnaire EQ-5D-5L and to quantify the pain in the affected joint with a Numeric Rating Scale (NRS). An informed consent was obtained from all participant.

Then, the lipoaspiration process and IA injection of AAT was performed in the operating theater with the patient under short, general anesthesia. These procedures as well as questionnaires were described in detail in our previous article [22].

Two physical therapists (JK and CB) were making a phone call to every single patient at a follow-up time (from 03.2019 to 04.2020), to fulfill exactly the same set of questionnaires. Moreover, the patients were asked to: (i) assess their satisfaction with the effects of the conducted treatment: from 0 (unsatisfied) to 10 (very satisfied), (ii) describe the rehabilitation, if it was performed (supervised or individual and duration in weeks) and (iii) indicate any additional treatment applied, like IA injections of hyaluronic acid (HA) or platelet- rich plasma (PRP), knee arthroscopy, partial or total knee arthroplasty (TKA).

After the collection of all of the data, we decided to exclude from the statistical analysis patients who had an IA injection of AAT combined with an arthroscopic debridement (4 patients), underwent any kind of the an additional treatment during a follow-up time (6 patients-TKA, 1 patient-AS debridement, 8 patients-IA injection of HA or PRP) and 1 patient with secondary knee OA after multi-ligamentous injury. Two patients were excluded because of the problems with communication caused by a coexisting mental illness. Finally, 37 participants were included in the statistical analysis (Table 1).

**Table 1:** Patients selection

→ 59	Patients who underwent IA injection of AAT
→ 22	Patients excluded from the study
	4–AAT injection were associated with AS debridement
	1–secondary knee OA after multiligamentous injury
	8–received additional IA injection with HA or PRP
	7–had to underwent surgical treatment (6 TKA, 1 AS debridement)
	2–with mental illness
→ 37	Included into statistical analysis
	31–bilateral IA injection (but only one joint was symptomatic)
	6–single joint IA injection

Statistical analysis was conducted with Statistica 12. Quantitative variables were presented with an average and a standard deviation. Shapiro–Wilk test was used to assess the normality of the distribution of data. *T* test was used to analyze the changes in the time for the paired data. In case of nonparametric distribution of the data, Willcoxon test or Mann–Whitney *U* test were used for the analysis. Kruskall–Wallis ANOVA and ANOVA univariate were used for the analysis of more than two samples. The correlation was checked with a Spearman rank-order correlation and if the correlation occurred, the univariate linear regression model analysis was conducted. ANOVA was used to find if the interaction effect occurred. The level of significance was set to *p* < 0.05.

## Results

The mean age of 37 participants (16 males and 21 females) included into the statistical analysis was 57.78 ± 7.39 years. The questionnaires were fulfilled after the average follow-up time of 27 ± 6.5 months. The patients presented I–IV knee OA stage in K-L scale (Table 2). In general, the mean BMI was 31.30 ± 7.51, 9 patients had ‘normal weight’ (BMI 18.5–24.9) and 28 patients had were ‘overweight’ or ‘obese’ (BMI ≥ 25).

**Table 2:** The demographic data

Total number of patients	37
Age (year)	57.78 ± 7.39
Gender	
Male	16
Female	21
Knee OA stage (Kellgren–Lawrence)	
I	1
II	9
III	20
IV	7
IA injection of AAT	
Bilateral	31
Unilateral	6
BMI (kg/m2)	31.30 ± 7.51
Follow-up time (months)	27 ± 6.5

The data represent the mean value ± the standard deviation

Both, the mean score in every C and a scale, improved at a follow-up time (Table 3). A significant difference (*p* < 0.05) compared with the baseline, was observed in pain [NRS], WOMAC, KOOS index, pain, symptoms, ADL, Sport and Rec, QoL, EQ-5D-5L Index.

**Table 3:** Total scores achieved in the questionnaires at the baseline and at the follow-up

	Score		*p* value
	Preoperative	Follow-up	
Pain [NRS]	4.95 ± 2.15	4.05 ± 2.01	0.0274^a^
EQ-5D-5L health state	70.35 ± 14.91	73.78 ± 14.26	ns^a^
EQ-5D-5L index	0.66 ± 0.15	0.73 ± 0.14	0.0077^a^
IKDC 2000	49.25 ± 8.75	51.39 ± 9.98	ns^b^
WOMAC	67.65 ± 18.75	75.06 ± 15.01	0.0026^b^
KOOS			
Index	58.60 ± 17.37	66.49 ± 16.05	0.0018^b^
Symptoms	64.17 ± 21.30	71.43 ± 19.27	0.0039^b^
Pain	63.01 ± 18.47	70.88 ± 16.24	0.0139^a^
ADL	67.87 ± 19.42	75.71 ± 15.10	0.0025^b^
Sport and Rec	26.76 ± 23.99	35.44 ± 28.40	0.0118^a^
QoL	39.53 ± 17.62	47.80 ± 21.51	0.0109^b^

The data represent the mean value ± the standard deviation. *ns* *p* value non-significant

^a^Analysis conducted with Willcoxon test

^b^Analysis conducted with *T* test; *ns* no significant changes observed, *p* value > 0.1

The satisfaction in the whole group was 6.16 ± 3.07. Due to the wide range of the satisfaction score values between patients, the subjects were divided into two groups: satisfied (satisfaction with the effects of treatment with AAT evaluated ≥ 7) and unsatisfied (satisfaction evaluated ≤ 6) with the effects of treatment. There was no significant difference between satisfied and unsatisfied patients in BMI and pain [NRS] at the baseline. 6 out of 7 patients with stage IV in K-L were unsatisfied with the effects of the treatment with AAT.

The results of the analysis of the influence of knee OA stage, BMI and satisfaction are presented in Table 4. Significant differences (*p* < 0.05) were found in pain [NRS], KOOS index, symptoms and QoL considering the influence of knee OA. In case of BMI, statistically significant differences (*p* < 0.05) were found in WOMAC, KOOS index, symptoms, pain, ADL. No differences were found between satisfied and dissatisfied participants.

**Table 4:** The influence of knee OA stage, BMI and satisfaction on results achieved in each score

	Knee OA stage [K-L]			*p* value	BMI		*p* value	Satisfaction		*p* value
	II (*n* = 18)	III (*n* = 40)	IV (*n* = 14)		Normal (*n* = 16)	Overweight/obese (*n* = 58)		Satisfied (*n* = 40)	Dissatisfied (*n* = 34)	
Pain [NRS]	3.67 ± 2.00	5.25 ± 2.01^a^	3.79 ± 1.72	0.0075^c^	3.94 ± 1.98	4.66 ± 2.14	ns^e^	4.33 ± 2.27	4.71 ± 1.93	ns^e^
EQ-5D-5L health state	78.17 ± 11.68	69.15 ± 15.78	71.43 ± 13.51	**0.0635**^**c**^	71.38 ± 17.15	72.26 ± 13.96	ns^e^	70.58 ± 14.13	73.82 ± 15.13	ns^e^
EQ-5D-5L index	0.74 ± 0.14	0.66 ± 0.13	0.69 ± 0.16	0.1062^c^	0.71 ± 0.19	0.69 ± 0.14	ns^e^	0.71 ± 0.15	0.68 ± 0.14	ns^e^
IKDC 2000	51.30 ± 10.11	49.25 ± 9.33	50.37 ± 8.26	ns^d^	51.87 ± 10.91	49.89 ± 8.97	ns^b^	50.65 ± 9.90	49.92 ± 8.86	ns^b^
WOMAC	77.84 ± 20.14	67.47 ± 16.03	72.54 ± 15.16	0.1140^c^	80.40 ± 16.67	68.86 ± 16.73	0.0169^b^	69.48 ± 19.32	73.56 ± 14.48	ns^b^
KOOS										
Index	71.00 ± 21.84	58.52 ± 13.91^a^	60.94 ± 14.53	0.0306^d^	70.83 ± 18.66	60.26 ± 16.04	0.0273^b^	62.48 ± 19.97	62.62 ± 13.18	ns^b^
Symptoms	79.56 ± 19.63	63.02 ± 19.63^a^	63.78 ± 19.20	0.0105^c^	76.53 ± 21.95	65.39 ± 19.60	0.0393^e^	70.00 ± 20.84	65.21 ± 20.09	ns^b^
Pain	74.71 ± 21.26	62.85 ± 15.09	67.12 ± 16.65	**0.0708**^**c**^	76.66 ± 16.64	64.27 ± 17.19	0.0122^b^	65.77 ± 21.05	68.33 ± 12.93	ns^e^
ADL	77.29 ± 21.19	68.44 ± 16.62	72.55 ± 15.10	ns^c^	79.66 ± 18.20	69.62 ± 17.11	0.0439^b^	69.66 ± 19.61	74.29 ± 15.11	ns^b^
Sport and Rec	44.72 ± 37.55	25.50 ± 19.57	26.07 ± 19.13	ns^c^	38.75 ± 34.18	28.99 ± 23.85	ns^e^	35.78 ± 30.27	25.59 ± 20.22	ns^e^
QoL	54.17 ± 24.54	40.00 ± 14.90^a^	37.05 ± 19.83^a^	0.0152^d^	50.78 ± 26.89	41.70 ± 17.38	ns^e^	45.00 ± 22.48	42.10 ± 16.74	ns^e^

^a^Significant difference in post hoc test in compare to stage II knee OA, *ns* no significant changes observed, *p* value > 0.1

^b^Analysis conducted with *T* test

^c^Analysis conducted with Kruskall–Wallis ANOVA

^d^Analysis conducted with ANOVA univariate

^e^Analysis conducted with the Mann–Whitney *U* test;

**Fig. 1:**
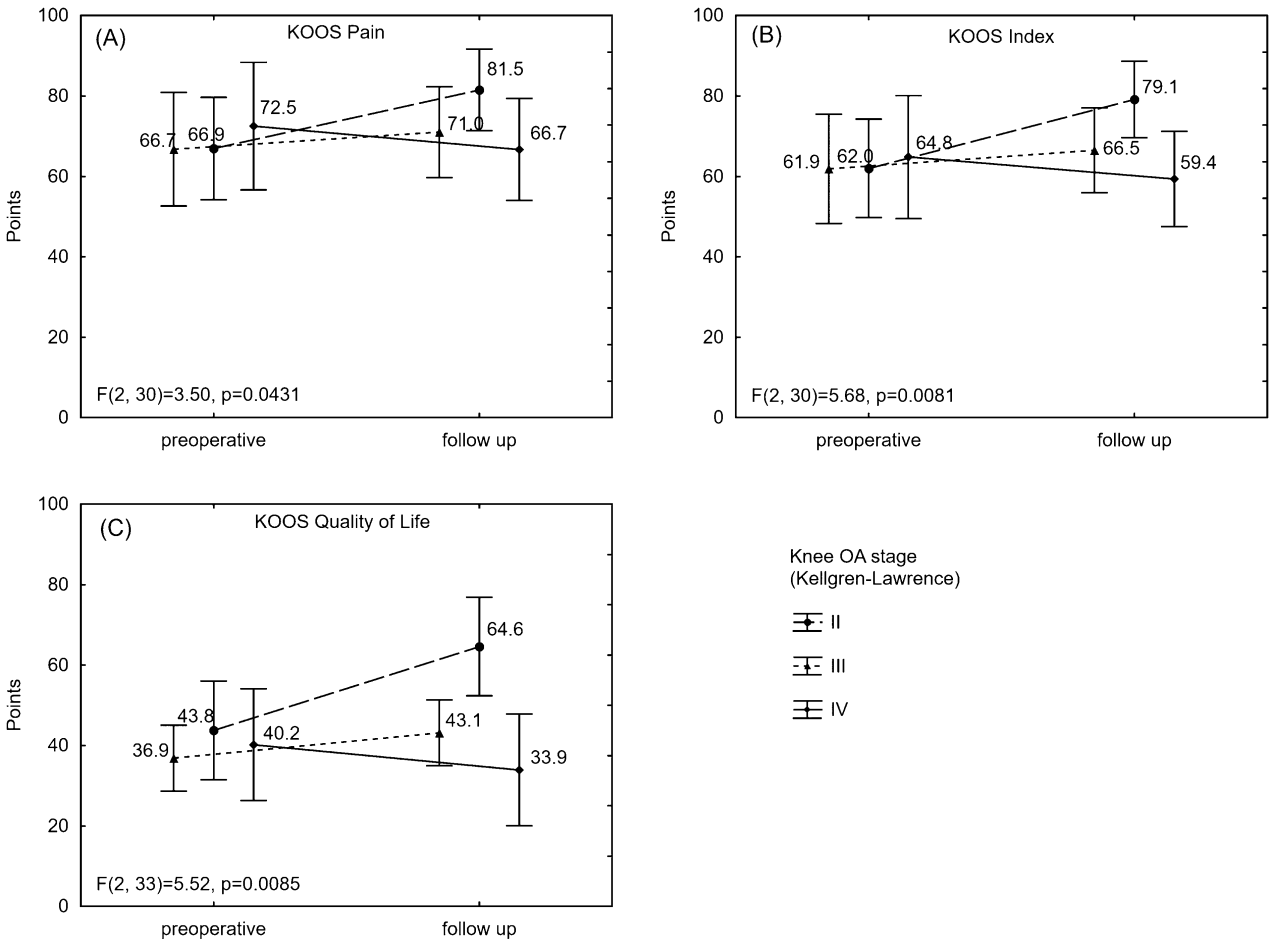
The interaction between the time and the stage of the knee OA in KOOS Index, pain, Qol. Results presented with an average and a confidence interval. *F* result of ANOVA

There was a significant interaction between the time and the stage of the knee OA in KOOS index, pain, QoL (Fig. 1) and a trend of the significance in KOOS symptoms (*p* = 0.0665). The participant with stage I in K-L scale was excluded from this analysis. In these scales and subscales, only group with II in K-L significantly improved. There was no significant interaction in pain [NRS], IKDC 2000, WOMAC, KOOS Sport and Rec and ADL and EQ-5D-5L scores. Regardless of the significance of interaction between the time and the stage of the knee OA, the patients with stage IV in K-L scale deteriorated in each score, except EQ-5D-5L. However, a small number of participants in all of three groups (K-LII—9 patients, K-LII—20 patients, K-LIV—7 patients) caused a wide range of standard deviation and a confidence interval in this analysis.

To analyze the correlation between the age and BMI and the changes in each questionnaire during a follow-up time, the difference between the final score and the baseline was calculated (Δ FU-preop). There was only one significant ‘weak’ correlation between BMI and EQ-5D-5L Health State (R Spearman = − 0.3080) and between age and EQ-5D-5L (R Spearman = − 0.3305) among all given scores. Then, the univariate linear regression model was conducted for BMI and EQ-5D-5L Health State as well as age and EQ-5D-5L Health State. The results were statistically significant, with *p* = 0.0356 and *p* = 0.062, respectively.

The rehabilitation was not taken into consideration because of considerable discrepancies among patients in a duration, frequency and a quality of exercises.

## Discussion

The main conclusion of this study is that the patients with stage II of the knee OA with normal BMI are most likely to benefit from IA injection of AAT, in contrast to the patients with stage IV, who will not be satisfied with the effectiveness of this kind of treatment. There were no adverse events reported at the donor site as well as in the treated knee joints.

When we have analyzed the data for the whole treatment group, that is all 37 cases, without categorization according to the OA progression stage, there was were no clinically significant improvements observed at the follow-up, although the results achieved the level of statistical significance. Such observation could lead to misleading conclusions, because, however, after dividing the patients into creating the subgroups, according to the stage of the disease, it was clearly visible that the patients with stage II of knee OA in WOMAC and KOOS achieved the significant clinical improvement. In the early stages of the Kellgren and Lawrence Scale, a minimally viable substrate can still be recognized: the required condition to generate the signaling pattern.

Hudetz et al. [23] treated 20 patients in late stage of knee OA (III and IV in K-L scale). They found statistical and clinical improvement after 12 months of follow-up in KOOS, WOMAC and Pain (VAS), although 3 patients (15% of the whole group) had to undergone TKA. Lapuente et al. [24] included 50 patients (100 knee OA joints) in the same knee OA criteria as Hudetz et.al [23]. They also found significant improvement in WOMAC and pain (VAS), but additionally they measured the satisfaction after the treatment with Spanish version of CRES-4 scale [25] and they found out, that subjects with stage IV in K-L scale expressed minor satisfaction than those with stage III (76.2% and 85.8%, respectively). In a recent study, Chachal et al. treated 12 patients with moderate‐to‐late‐stage knee OA with autologous bone marrow-derived MSCs but did not observe improvements in cartilage morphology at 12 months, based on MR imaging [26]. We have observed similar effect in our studies with IA-AAT: in our group, over 27 months of posttreatment, 15 of 55 subjects (27%) treated with AAT underwent additional treatment. Mean satisfaction in treated group was 6.16 (± 3.07) and 6 out of 7 patients with stage IV evaluated the satisfaction as less than 7 in 10 points. Such lack of regenerative effects may be indicative of changes in water retention, changes in interaction between collagen and water, or changes in the normal orientation of the collagen fibrils. Those results clearly indicate that IA injections of AAT is was not very effective for enrolled patients with severe knee OA or that patients’ expectations before conducting the treatment are were too high and it is good to inform them that hampering progression of the disease should be considered as a positive result of the treatment.

Several studies have tested the efficacy of knee OA treatment with IA injection of AAT so far, but only one of them was concentrated on defining the most suitable patients in terms of the radiographic changes, pain level, age or BMI [27]. Schiavonne Panni et al. [27] preceded IA injection with arthroscopic debridement in 52 patients with early knee OA (K-L 0-II). Patients were assessed retrospectively with the International Knee Society (IKS) knee and function scores and VAS, with an average follow-up of 15.3 months (6–24 months range). They observed a significant improvement in every score at follow-up. However, patients with preoperative VAS 8 and higher showed significantly greater percent improvement in IKS and pain scores than those who presented VAS below 8 at the baseline. Moreover, authors observed an immediate mechanical effect of adipose tissue transfer, probably based on lubricating capacity of fat tissue, which helped to restore a range of motion and function. 50 out of 52 patients (96.2%) declared their satisfaction with the effects of an applied treatment. In our study, 20 out of 37 patients were satisfied, but in this intervention group, there was no patient with K-L 0 and only 1 patient with K-L I and nobody received arthroscopic debridement before IA injection of AAT.

MSCs are cells which sense the pro-inflammatory environment of defected tissues inside the affected joint and answer with secretion of bioactive molecules, like IL- 1Ra, IL- 4, IL- 10, prostaglandin 2 and TGFβ or mediating by direct cell- cell contact [13, 14, 18, 21, 24, 28]. These substances are responsible for an inhibition of the production of TNFα and IL-12 by macrophages and inhibition of a proliferation and activation of T lymphocytes, B lymphocytes, NK (Natural Killer) cells and neutrophiles [18, 24]. These events result in restoration of homeostasis, due to an increase of anti-inflammatory and decrease of pro-inflammatory molecules [19, 29]. For the entire joint environment, this means slowing progression of degeneration.

High value of BMI is a very important risk factor in primary OA [3, 4, 7, 30]. Biomechanically, obesity causes an increase of load on the joints, which leads to earlier initiation and faster progression of this disease [31]. Additionally, visceral fat tissue secretes more pro- inflammatory molecules than subcutaneous one [32]. This phenomenon explains why non- weight bearing joints are also more prone to OA in patients with higher BMI and why adipose tissue loss is more effective in symptomatic relief in knee OA than the loss of the body weight [33]. In our study, there was only one correlation between the improvement in questionnaires and BMI, but 5 out of 7 participants with stage IV of knee OA had BMI above 25, moreover every patient who should have underwent additional treatment over follow-up time had BMI above 25 as well.

## Conclusion

The current retrospective study evaluated the effects of intra-articular autologous adipose tissue injections in a cohort of 37 patients with knee OA. Based on the outcomes, The patients who is the most likely to benefited from the knee OA treatment with an intra- articular injection of an autologous adipose tissue has to presented OA stage II in K-L scale. Moreover, we have noticed It is an important need to inform the patients about a limited capabilities of AAT before conducting the treatment, to avoid dissatisfaction.

## Data Availability

The datasets used during the current study are available from the corresponding author on reasonable request.

## Abbreviations

OA: Knee osteoarthritis
MSCs: Mesenchymal stem cells
PRP: Platelet rich plasma
NSAIDs: Non-steroidal anti-inflammatory drugs
KOOS: The Knee injury and Osteoarthritis Outcome Score
IKDC 2000: International Knee Documentation Commitee 2000
WOMAC: Western Ontario and McMaster Universities Osteoarthritis Index
EQ-5D-5L: Health Questionnaire
TUG: The timed up and go test
5 x STS: The 5 times sit to stand test
10mWT: The 10m walk test
HA: Hyaluronic acid
